# Delta central venous pressure and dynamic indices of preload in postsurgical ICU patients

**DOI:** 10.1186/cc9469

**Published:** 2011-03-11

**Authors:** M Cecconi, F Caliandro, E Barbon, V Elliott, A Dewhurst, A Rhodes

**Affiliations:** 1 Saint George's Hospital, London, UK

## Introduction

Pulse pressure variation (PPV) and stroke volume variation (SVV) are indices of fluid responsiveness. We tested whether delta central venous pressure (δCVP) could be used to see if enough volume has been given in order to produce a response in SV and therefore improve the accuracy of PPV and SVV [1].

## Methods

Forty-nine fully ventilated patients in sinus rhythm were admitted postoperatively to the ICU monitored with pulse power analysis (PulseCO; LIDCO, Cambridge, UK). Fluid challenge (FC) consisted of 250 ml colloid over 5 minutes. Responder: SV increase >10%. δCVP was used to define two groups of patients: A (δCVP 0 to 1 mmHg) and B (δCVP >2).

## Results

Eighty-two FCs were performed. There were 33% responders in A versus 36% in B (not significant). For A + B, SVV and PPV AUCs were 0.81 and 0.78. There was no statistically significant difference in the AUC for SVV and PPV between A and B, but there were different best cut-off values (Table 1).

**Table 1:** 

All patients	AUC groups A + B, best cut-off groups A + B	AUC group A, best cut-off group A
SVV	0.81 (SE 0.06), 11.5% (77%/76%)	0.89 (SE 0.07), 15.5% (88%/88%)
PPV	0.78 (SE 0.06), 13.5% (75%/76%)	0.84 (SE 0.09), 15.5% (81%/75%)

## Conclusions

Our data suggest that SVV/PPV efficacy in predicting a fluid response cannot be improved by looking at δCVP. More patients are needed to investigate the relationship between δCVP and best cut-off values for SVV and PPV.
